# Medical News and Miscellany

**Published:** 1888-05

**Authors:** 


### Medical News and Miscellany.

REMOVALS.—Dr. S. L. Post, from Alvarado to Gainesville; Dr. W. W. Wallace, from San Augustine to Abilene; Dr. J. P. Lehde. from New Braunfels to Austin; Dr. J. B. Kilpatrick, from Celina to Commerce; Dr. William Pearson, from Cleburne to Joshua; Dr. M. S. Howell, from Farmersville to Lien.

DR. S. H. STOUT, of Cisco, met with a serious accident last month; he fell and dislocated his shoulder joint. By resolution, a telegram was sent him, and also one to Dr. Clopton, by the State Association in convention, expressing sympathy.

DIED.—Dr. H. J. Hunter, of Palestine, died May 4th, aged 50. Graduate N. O. School Med., 1860.

DR. W. F. BUCK, late of Pecos, lost his wife and two young daughters last month, within a few weeks. The doctor's own health is much impaired, and he has removed temporarily to Salem, Lee County, Ala. He has our sympathy in his misfortune.

DR. F. W. KAISER, of Flatonia, who was expelled from the State Association last year, for associating himself in practice with Dr. Johnson, a "legal practitioner," but without a diploma, was re-admitted to membership, and Dr. Johnson, having meantime graduated M. D. at Tulane University, was also elected to membership at the Galveston meeting.

At the New York Polyclinic.—Dr. C. C. Black, of Round Rock, is at the Polyclinic. He writes that he sees Daniel's Texas Medical Journal on every desk, and feels that he has met an old friend whose society is very congenial and pleasant.

The Texas Medical College at Galveston is an assured fact. It is understood that when under headway, and when the Medical Department of the University of Texas becomes able to begin, the two are to be merged, i. e. the Texas Medical College is to turn over all the property to the University Regents, and a new Faculty will be organized. Thus, in time, the Medical Department of the University will receive the benefit of the Seely donations and the city donation—aggregating one hundred thousand dollars—but the present organization has nothing to do with the $50,000 appropriated by the legislature for the University Medical School.

We learn that at the meeting of the Trustees of the Texas Medical College recently held, the following appointments were made:

Professor of Science and Art of Surgery, B. E. Hadra, M. D., of Austin.

Professor of Obstetrics, etc., J. F. Y. Paine, M. D., of Galveston.

Professor of Anatomy, A. W. Fly, M. D., of Galveston.

Professor of Theory and Practice of Medicine, H. C. West, M. D., of Galveston.

Professor of Physiology and the Institutes of Medicine, H. P. Cook, M. D., of Galveston.

Professor of Materia Medica and Therapeutics, E. Randall, Jr., M. D., of Galveston.

Lecturer of Deseases on the Eye, Ear and Throat, G. P. Hall, M. D., of Galveston.

Demonstrator of Anatomy, Geo. H. Lee, M. D., of Galveston.

The preliminary course will begin October 15, 1888, and the Regular Session November 1. The Trustees have leased the City Hospital buildings for ten years and the Faculty will be the Hospital Staff. Clinical material will be abundantly supplied. Our best wishes attend this noble enterprise.

---

PERSONAL.—Dr. Wm. M. Yandell, whose paper appears in this issue, is a son of the late Professor Lunsford Yandell, Sr., of Louisville, Ky. His paper bore off first prize at the Gross Medical College Commencement, for "best Essay on any subject connected with medical jurisprudence," Judge Campbell making the award—a most appropriate one—two handsome volumes, "Autobiography of Sam'l D. Gross." Dr. Yandell has settled in El Paso.

---

TIME !—One more issue of the JOURNAL will complete Vol. 3, and the third year of its unprecedented success. We can but feel grateful to those who have contributed to that success, by pen and pocket; who have paid their subscription promptly in advance, and have as promptly renewed. We call on them now to renew their subscriptions—many have already done so,—for we need the money as badly as we ever did. But what shall we say of those few who have not paid at all?—who have received and read and enjoyed and profited by our and others' labors, one whole year—two—*three* whole years—and who turn a deaf ear to polite solicitations for payment, and *get mad* at a dun? Well, we suppose the best thing for us and for the JOURNAL would be to drop them, and lose four or six dollars, rather than let them take it for nothing the balance of life; but, the *law* says a subscriber cannot be dropped so long as he is in arrears! How about that? Now we do hope *somebody* will take a hint and hustle up the amount due us; Vol. 3 is *nearly* out, and it is much easier to pay $2 or $4, or even $6, than

$8 or $10; and then—we need it; it is a kind of *must* with us, and some of the bills are "kinder" *musty*. We do not want to advertise them for sale, like Lanphere did; for thanks, we have not enough of the $4 and $6 kind to justify it; but, something must be done with them. Meantime, now is the time to come to time, to pay up, to renew, to subscribe. Get up clubs at $15 for ten, and a copy free to sender, and let them rattle in as they did at Galveston and since. Fifty-four new *volunteer* subscribers since the meeting.

---

### STRAINING AT A GNAT AND SWALLOWING A CAMEL.

[The following clipping is sent to us by one of our largest advertisers, manufacturers of well-known and useful pharmacals, and we produce it with pleasure.—Ed.]

"American medical, pharmaceutical and trade journals, usually keen to detect a hidden advertisement in communications recommending new drugs and preparations when the same emanate from home sources, throw caution and ordinary business sense to the winds when it comes to recommending and puffing the very same class of merchandise, bearing a foreign name and recommended by foreign authority. The success of one or two German chemicals, the products of synthesis, opened the doors for a flood of antiseptins, antifebrins, antipyrins, and other "antis" ending in "ol" or "in." They come to us covered all over with patents—patents covering the names, the process of manufacture, the ingredients (save those which are kept absolutely secret), the modes of dispensing, the package, the label—in short, everything that a patent can be made to cover. In a word, they are patent medicines in the very widest and strictest sense of the term; and yet they are received with enthusiastic welcome by the press and practioner, and are given, gratis and gladly, advertisements that money could not purchase for a home product, even though ten times more valuable, and not one-tenth so much patented.

"One of the proprietors of a drug of this sort, recently established in America, on being approached by the solicitor of advertising for an American medical journal, answered very curtly that "they didn't have to advertise their article. They got all the advertising they wanted, for nothing, in the shape of laudatory communications in the reading matter of the medical journals." Which was true, every word of it, and that in spite of the fact that it was a patent medicine. The very journal for which the agent was soliciting, and in the very copy which he carried as a specimen, contained no less than six laudatory notices of the drug in question—one of them a communication covering several pages and herald-its virtues in almost every known form of disease.

"Per contra, the same journal had enjoyed for years a handsome revenue from the advertisement of a reputable proprietary medicine house of this city, but had persistently refused to admit within its reading matter a little notice commendatory of one of its specialties, the formula for which was printed on every bottle.

"It is useless to plead that these imported patents are so valuable that the profession *must* have them and *must* use them, secret nostrums though they be. This is not true, nor is it true that the manufacturers over there are any more honest or frank as to the nature and origin of their wares than are the American manufacturers of similar drugs. In proof of this assertion we call the attention of our readers to Gawalowski's merciless exposure of a new compound which is getting ready in Germany to make a descent on Europe and America in the style of its predecessors—the antiseptic kreolin, of the wondrous value of which the advance guard of certificates have already commenced to appear in our journals. Will the latter be warned in time, or will they swindle themselves out of thousands of dollars by giving it the usual American welcome and gratis advertising?"—Dr. James' editorial in St. Louis Druggist.

